# Integrating GWAS and gene expression data for functional characterization of resistance to white mould in soya bean

**DOI:** 10.1111/pbi.12918

**Published:** 2018-05-07

**Authors:** Zixiang Wen, Ruijuan Tan, Shichen Zhang, Paul J. Collins, Jiazheng Yuan, Wenyan Du, Cuihua Gu, Shujun Ou, Qijian Song, Yong‐Qiang Charles An, John F. Boyse, Martin I. Chilvers, Dechun Wang

**Affiliations:** ^1^ Department of Plant, Soil and Microbial Sciences Michigan State University East Lansing MI USA; ^2^ Department of Biological Sciences Fayetteville State University Fayetteville NC USA; ^3^ Department of Horticulture Michigan State University East Lansing MI USA; ^4^ Soya bean Genomics and Improvement Laboratory United States Department of Agriculture Agricultural Research Service Beltsville MD USA; ^5^ USDA‐ARS Plant Genetics Research Unit at Donald Danforth Plant Science Center Saint Louis MO USA

**Keywords:** *Sclerotinia sclerotiorum* (Lib.) de Bary, GWAS, RNA‐seq, soya bean (*Glycine max* (L.) Merr.), single nucleotide polymorphism

## Abstract

White mould of soya bean, caused by *Sclerotinia sclerotiorum* (Lib.) de Bary, is a necrotrophic fungus capable of infecting a wide range of plants. To dissect the genetic architecture of resistance to white mould, a high‐density customized single nucleotide polymorphism (SNP) array (52 041 SNPs) was used to genotype two soya bean diversity panels. Combined with resistance variation data observed in the field and greenhouse environments, genome‐wide association studies (GWASs) were conducted to identify quantitative trait loci (QTL) controlling resistance against white mould. Results showed that 16 and 11 loci were found significantly associated with resistance in field and greenhouse, respectively. Of these, eight loci localized to previously mapped QTL intervals and one locus had significant associations with resistance across both environments. The expression level changes in genes located in GWAS‐identified loci were assessed between partially resistant and susceptible genotypes through a RNA‐seq analysis of the stem tissue collected at various time points after inoculation. A set of genes with diverse biological functionalities were identified as strong candidates underlying white mould resistance. Moreover, we found that genomic prediction models outperformed predictions based on significant SNPs. Prediction accuracies ranged from 0.48 to 0.64 for disease index measured in field experiments. The integrative methods, including GWAS, RNA‐seq and genomic selection (GS), applied in this study facilitated the identification of causal variants, enhanced our understanding of mechanisms of white mould resistance and provided valuable information regarding breeding for disease resistance through genomic selection in soya bean.

## Introduction

*Sclerotinia sclerotiorum* (Lib.) de Bary has a broad host range and is documented to infect at least 408 plant species (Boland and Hall, 1994). On soya bean, *S. sclerotiorum* causes the disease Sclerotinia stem rot that also known as white mould. It causes yield loss through the reduction of seed number and weight as well as seed quality (Hoffman *et al*., 1998). The pathogen can persist in the field through the production of sclerotia, a resting body for the fungus. Additionally seeds can be infected and act as a source of inoculum particularly to noninfested fields (Danielson *et al*., 2004; Yang *et al*., 1999). In 1994, 2004 and 2009, it ranked second to soya bean cyst nematode on total yield lost in US soya bean production (Koenning and Wrather, 2010; Wrather and Koenning, 2006; Wrather *et al*., 1997).

Fungicide management of white mould can be difficult to achieve, and complete control is not possible, with reductions in disease incidence ranging from 0 up to 60% (Peltier *et al*., 2012). To reduce inoculum and create unfavourable conditions for fungal and disease development, several agronomic practices such as reduced tillage and crop rotation have been suggested (Kurle *et al*., 2001; Peltier and Grau, 2008; Workneh and Yang, 2000), but none of them has been completely effective. Host plant resistance is the most economical and environmental friendly way of controlling soya bean white mould incidence to prevent yield loss. Although no soya bean cultivars with complete resistance to white mould have been developed through conventional breeding, soya bean plant introductions (PIs) and varieties showing differences from susceptible to partially resistance to the pathogen have been reported (Chen and Wang, 2005; Kim *et al*., 1999). It is important for breeders to understand the genetics of resistance available in soya bean germplasm to develop varieties with greater resistance.

Quantitative trait loci (QTL) mapping in bi‐parental derived population is a method commonly used to dissect the genetics basis of white mould resistance in soya bean. The previous mapping studies have identified a total of 103 QTLs (http://www.soybase.org/), which distributed on 17 chromosomes (LGs) of soya bean. Among these QTLs, only six loci were identified under field conditions (Huynh *et al*., 2010; Kim and Diers, 2000) and the rest were identified under greenhouse or growth chamber studies with various artificial inoculation methods (Arahana *et al*., 2001; Guo *et al*., 2008; Vuong *et al*., 2008). Unfortunately, these tests under controlled conditions produced a poor correlation with the resistance observed in the field (Guo *et al*., 2008; Nelson *et al*., 1991). Moreover, such inoculation techniques cannot be used for large‐scale application in the field. It is probably because different isolates, inoculation techniques and resistance sources were used, most of those QTLs showed limited reproducibility. Therefore, there is still a great need to map and identify white mould resistance genes in soya bean.

A large‐scale shotgun sequencing of *Glycine max* var. Williams 82 (2*n*=40) began in the middle of 2006 and was completed early in 2008. Approximately 978 million base pair (Mb) is captured in 20 chromosomes, with a small additional amount of mostly repetitive sequence in unmapped scaffolds (Schmutz *et al*., 2010). With the advent of high‐throughput genotyping technologies, such as resequencing and microarray, GWAS has become an affordable and powerful tool for dissecting complex traits in soya bean. To date, GWAS has been performed for the dissection of soya bean traits, such as disease resistance (Bao *et al*., 2014; Han *et al*., 2015; Wen *et al*., 2014), yield, protein and oil content in soya bean (Hao *et al*., 2012a; Hwang *et al*., 2014; Sonah *et al*., 2014; Wen *et al*., 2015). As for white mould, a GWAS identified three genomic regions related to resistance on a panel of 101 soya bean PIs screened under controlled conditions. The strongest association was found on Chromosome 3 (Iquira *et al*., 2015). With a germplasm panel of 130 breeding lines from eastern Canada, the same research group found that the strongest association switched to Chromosome 15 and that none of the QTLs identified in these two association studies overlapped (Bastien *et al*., 2014). Additionally, a GWAS was conducted to identify loci associated with stem pigmentation, an indicator of resistance to white mould, in 330 diverse soya bean landraces; a major QTL on Chromosome 13 were identified as associated with stem pigmentation (Zhao *et al*., 2015). Despite these results, GWAS does not necessarily lead directly to the gene(s) at a given locus because of insufficient marker density and linkage disequilibrium. This raises the question of whether GWAS data sets can yield additional insights when combined with other data modalities. Recently, interrogating the significant SNPs identified from GWAS for associations with gene expression data (Hao *et al*., 2012a,b; Hernandez *et al*., 2012) has been employed to interpret GWAS results.

With this background in mind, two diverse panels consisting of 405 soya bean PIs and 905 improved lines were evaluated for response to white mould in greenhouse and field environments. With employing high‐density SNP genotyping data and RNA‐seq data, our study aimed (i) to identify loci associated with resistance to white mould via GWAS, (ii) to explore candidate genes located at GWAS‐identified loci through differential expression analyses and (iii) to assess the potential of marker‐based prediction model as a new approach in soya bean breeding. We believe that genetic dissection in two different germplasm panels will provide complementary information for understanding of mechanisms underlying white mould resistance.

## Results and discussion

### Phenotypic characterization of the two panels

Greenhouse evaluations of the two panels of germplasm for resistance to white mould revealed a broad range of resistance levels (Table 1). As a mycelial inoculation method was used to assess the resistance level of each line in a greenhouse under the conditions facilitating disease development, severe disease symptoms were observed across all greenhouse trials. As can be seen in Figure S1, the distribution of mortality data was skewed towards susceptible. However, live node (un‐infested node) number covered a broad range (0 to 4) with normal distribution in both panels. Resistant check AxN‐1‐55 showed more live nodes than the average (1.67 and 2.0), whereas the susceptible check Olympus developed much longer lesions with no live nodes remaining (Figure S1).

**Table 1:** Descriptive statistics, ANOVA and broad‐sense heritability of disease indexes in the two panels

Environment	Population	Min.	Max.	Mean	Std.^b^	*G* c	*G* × *E* d	*H*
Field (DSI)	PIs (279^a^)	0.0	80.7	31.2	17.7	e	e	0.51
	Improved lines (421)	3.2	77.9	30.8	15.2	e	e	0.63
Greenhouse (No. of live node)	PIs(405)	0.0	4.8	1.7	0.96	e	ns	0.52
	Improved lines (915)	0.0	5.0	2.0	1.10	e	e	0.69

ns, not significant; *H,* broad‐sense heritability.

a No. of accessions.

b std., standard deviation.

c *G*, Genotype across different environments.

d *G* × *E*, Genotype × Year.

e Significant at *P *<* *0.01.

In field tests, averaged over 2 years, a large variation in white mould resistance was also observed across assayed soya bean accessions in both panels. Disease severe index (DSI) had a mean of 31.2 and 30.8 for PIs and improved lines, respectively, with more than a 20‐fold difference among the resistant and susceptible lines (Table 1, Figure S2). ANOVA for the two disease indices, field derived DSI and greenhouse derived number of live nodes, indicated that the factors of accession, year and accession by year had significant effects (Table 1). The broad‐sense heritability of DSI was 0.63 and 0.51 for improved lines and PIs, respectively, suggesting that genetic variability may still play a substantial role in white mould resistance under significant *G* × *E*.

A previous study demonstrated that maturity groups (MGs) significantly affected disease incidence (Yang *et al*., 1999). In the present study, we did find negative correlation between maturity and DSI. However, the correlation was insignificant and likely was due to limited coverage of MGs (MGI to MG III) among the tested lines. Nevertheless, there were significant (α = 0.05) and positive correlations between lodging and DSI in field trials for both panels. Significant correlations were also observed for DSI between the 2014 and 2015 field trials for both panels. Meanwhile, DSI had a lower (*r *=* *−0.22 in 2014, *r *=* *−0.12 in 2015) but statistically significant correlation (*P *<* *0.05) with live node number measured in the greenhouse for improved lines (Table S1). No statistically significant correlation was observed between DSI and live node number for PIs (Table S1).

### Polymorphic marker, patterns of linkage disequilibrium and profile of population structure

Profiles of 52 041 SNPs were characterized in 405 soya bean landraces and 915 improved lines with SoySNP50K BeadChip. After quality control, a total of 31 600 and 35 708 SNPs passed the filters and were used in linkage disequilibrium (LD) analysis and GWAS for the improved lines and PIs, respectively. Moreover, population structure analysis was based on 4549 SNPs with minor allele frequency (MAF) >20% and physical distance >60 kb.

**Figure 1:**
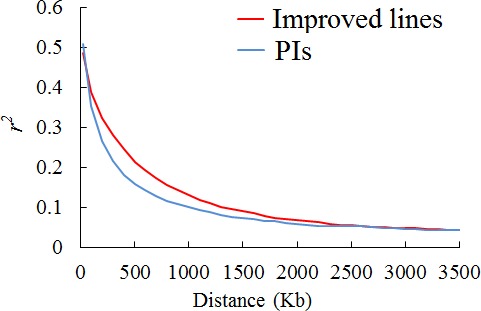
Genome‐wide average LD decay in two subsets of improved lines and PIs. Decay of LD (measured as genotypic *r* ^2^) as a function of distance between SNPs.

As the decay of LD and population structure of the two panels were characterized in our previous published paper (Wen *et al*., 2015), herein we conducted the corresponding analysis for the subsets of the two panels used in field trials. Decay of LD over increasing physical distance is illustrated in Figure 1. The LD rate, measured by *r* ^*2*^ declining to half its maximum value, was 240 kb and 370 kb in the two subsets of PIs and improved lines, respectively. These LD decay estimates are larger than previously published values in landraces of 187 kb and in improved lines of 233 kb (Wen *et al*., 2015). This difference may be attributed to curtailing of sample size in this study, as a similar phenomenon was observed in maize (Yan *et al*., 2009). The estimates of LD decay herein suggest at least 2700 (1000 Mb/370 kb) to 4200 (1000 Mb/240 kb) markers will be needed for whole genome scanning in soya bean, as the soya bean genome is known to extend slightly over 1000 Mb. The number of polymorphic markers in both panels exceeds 30 000, which ensure the coverage of most LD blocks and a reasonable power to identify common variants of large effect associated with white mould resistance. Note that LD decay varies across different chromosomes, and particularly within heterochromatic or euchromatic chromosome regions. Our previous study demonstrated a large variation in extent of LD among chromosomes with a range from 100 kb to 430 kb (Wen *et al*., 2015). Moreover, Hwang *et al*. (2014) identified that LD decay rate in heterochromatic and euchromatic chromosome regions was 360 kb and 9600 kb, respectively.

**Figure 2:**
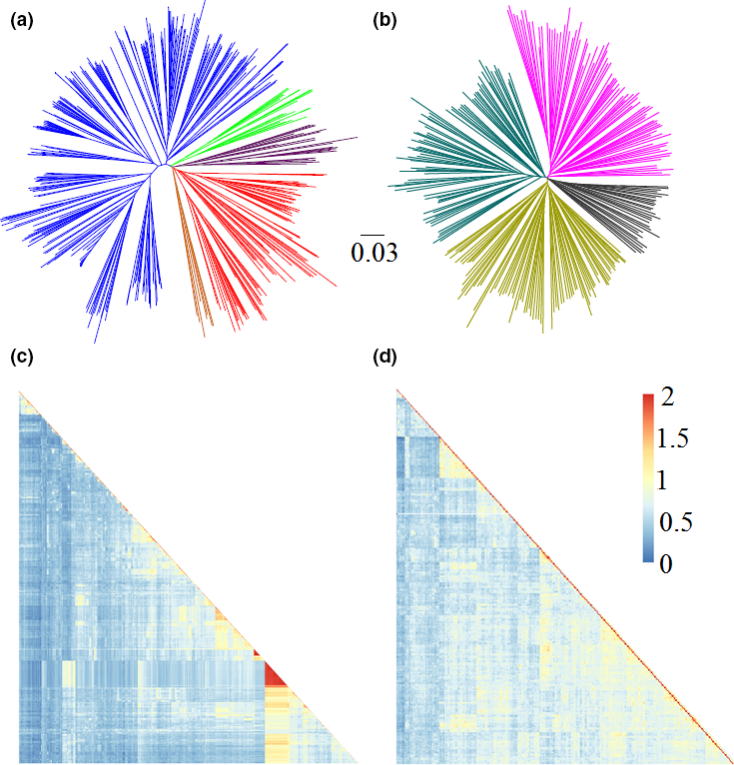
Population structures and kinship heat map of two subsets of soya bean PIs and improved lines. (a) NJ tree of soya bean improved lines. The five subgroups identified from the tree are colour‐coded. (b) NJ tree of soya bean PIs. The four subgroups identified from the NJ tree are colour‐coded. (c) A heatmap of the kinship value among accessions of the improved lines. (d) A heatmap of the kinship value among accessions of PIs.

As population structure can result in spurious associations, it has constrained the use of association studies in human and plant genetics (Yu *et al*., 2006). Neighbour‐joining (NJ) cluster analysis was performed on the two subsets to explore the relatedness among the sampled accessions. As for the NJ tree, no clear grouping was observed among PIs, whereas a few genotypes from the improved lines showed close relatedness and subtle grouping trends (Figure 2). These results indicate a lower level of population structure in PIs than that in improved lines. The chi‐square test was used to test whether the SNP data‐based subgroups were associated with geographic origins or MGs (Table S2). The results showed very significant association (*P *<* *0.0001) between the two grouping factors. For example, PIs from Japan were mainly (63%) clustered in Cluster 4, whereas Cluster 1 contained 31 accessions, of which 19 were from northern China; improved lines belonging to MG II dominated Cluster 4, whereas Cluster 3 contained eight accessions, of which all were from MG III (Table S2). These results show population structures positively correlated with geographic origins, which validated the previous analyses (Hao *et al*., 2012a,b; Wen *et al*., 2014, 2015) and provide additional insights into the fine‐scale patterns of ancestry resulting from geographic differentiation and regional soya bean breeding efforts. Taken together, these results highlight the need to account for population structure when conducting association analyses in soya bean.

### GWAS for white mould resistance

**Figure 3:**
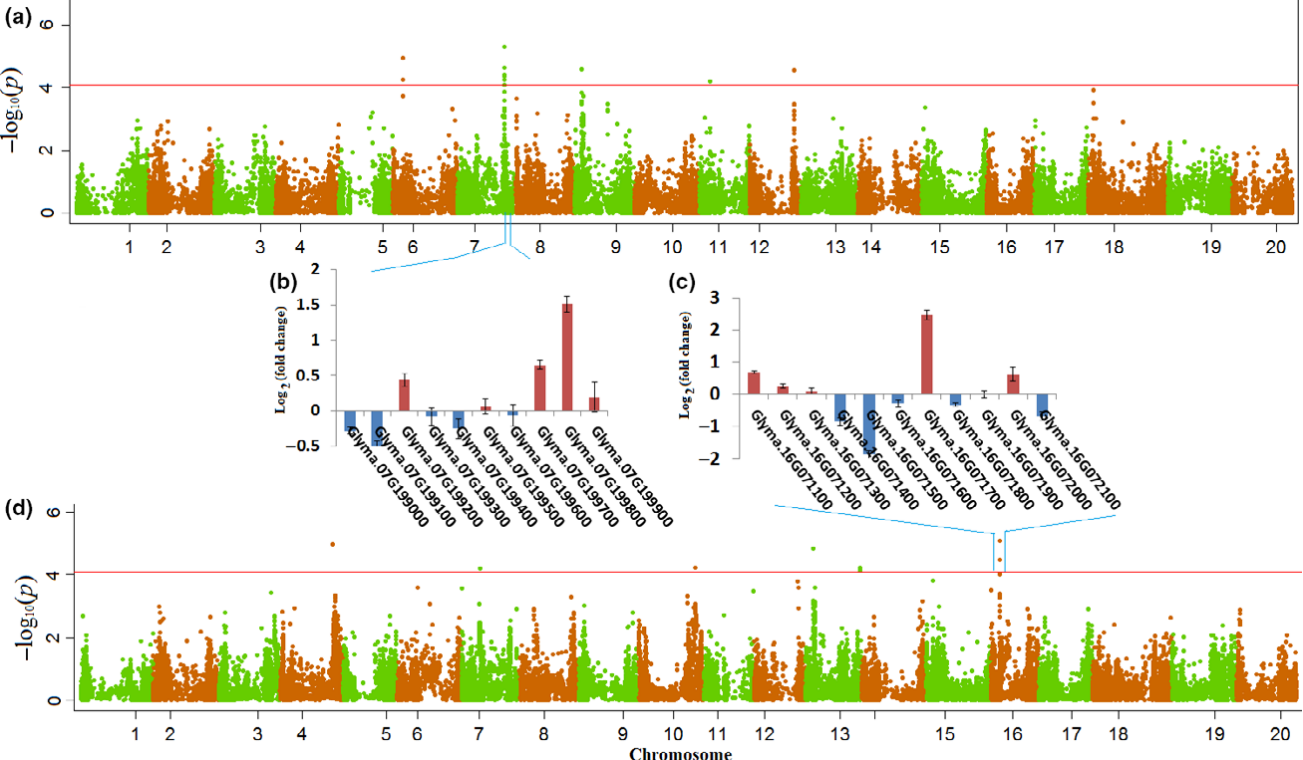
Visualization of the GWAS results in the two association panels and changes in transcript level of genes around peak SNP. (a) Manhattan plots of the MLM for live node in PIs. The − log10 *P*‐values from a genome‐wide scan are plotted against the position on each of the 20 chromosomes. The horizontal red line indicates the genome‐wide significance threshold (FDR <0.05). (b, c) Transcript‐level difference in candidate genes between SSR partially resistant and susceptible genotypes measured by comparisons of Log_2_ (fold change) of FPKM around peak SNP. (d) Manhattan plots of the MLM for live node in improved lines.

GWAS was conducted using the phenotypic variation data from greenhouse and field trials in a mixed linear model (MLM), which accounts for both population structure (top four principal components) and familial relatedness (*K* matrix). The MLM model resulted in a good approximation to expected cumulative distribution of *P* value (Figure S3). A total of 21 SNPs significantly associated with the number of live nodes were identified (Table 2 and Figure 3) from the greenhouse evaluations. Given that some of these SNPs showing strong LD with each other and could not be considered as separate loci, all of these SNPs were clumped using LD block as a criterion to define major QTL. After the clumping of SNPs, 11 significant loci scattered across nine chromosomes were identified (Table 2). The peak SNPs at the identified loci explained approximately 24.6% and 22.1% of the total phenotypic variance in the improved lines and PIs, respectively. In the panel of improved lines, the locus with the largest effect (*R* ^2^ = 5.1%) comprised four SNPs covering 44.5 kb around 7.2 Mb on Chromosome 16. In the panel of PIs, the locus most significantly associated (*P* value = 4.7 × 10^−6^) with number of live nodes comprised six SNPs covering 270 kb at 36.7 Mb on Chromosome 7.

**Table 2:** SNPs significantly associated with white mould resistance and a subset of candidate genes identified by RNA‐seq from greenhouse trials

Panel	Loci	SNP	Chr.	Position^a^	*P*	Allele	*R* ^2^ (%)	QTL^b^	Subset of candidate genes^c^ based on RNA‐seq			
									Name	Annotation	Log_2_(fold change)	TP^d^ (hpi)
Improved lines	1	ss715588043	4	44059284	1.1 × 10^−5^	A/G	4.0		Glyma.04G184400	F‐box only protein	1.5	12
	2	ss715596204	7	10951353	6.1 × 10^−5^	A/G	3.6	1‐2	Glyma.07G109600	SBP domain	1.7	12
	3	ss715607404	10	44648970	6.0 × 10^−5^	C/A	3.2		Glyma.10G214500	Unknown	1.6	12
	4	ss715616839	13	15951647	1.4 × 10^−5^	A/G	3.8		Glyma.13G062000	NAM protein	−2.0	48
	5	ss715616533	13	44344336	7.1 × 10^−5^	A/G	4.1		Glyma.13G355600	NAD‐dependent epimerase	1.7	12
		ss715616535	13	44357080	5.9 × 10^−5^	T/C	4.2					
	6	ss715625406	16	7257702	9.1 × 10^−5^	T/C	4.1		Glyma.16 g071700	LOB domain containing	2.5	12
		ss715625408	16	7265131	8.1 × 10^−6^	C/T	5.2					
		ss715625410	16	7272893	3.3 × 10^−5^	T/G	4.5					
		ss715625414	16	7302240	9.7 × 10^−5^	A/G	4.0					
PIs	7	ss715595608	6	8486465	5.6 × 10^−5^	T/C	4.7					
		ss715595609	6	8488833	1.1 × 10^−5^	T/G	4.6		Glyma.06G107800	Serine hydroxyl methyltransferase	1.8	12
	8	ss715597461	7	36664586	2.2 × 10^−5^	C/T	5.1					
		ss715597466	7	36679589	3.6 × 10^−5^	C/T	4.9					
		ss715597467	7	36684209	4.1 × 10^−5^	A/G	4.8					
		ss715597472	7	36740564	4.7 × 10^−6^	T/C	5.0		Glyma.07G199800	MAC/Perforin domain	1.5	48
		ss715597474	7	36745679	5.6 × 10^−5^	T/C	4.8					
		ss715597504	7	36936795	7.9 × 10^−5^	T/C	3.7					
	9	ss715605211	9	5948655	2.6 × 10^−5^	C/T	4.3	1‐3	Glyma.09G062100	LRR	2.8	12
	10	ss715611206	11	8151411	6.4 × 10^−5^	T/G	3.8	3‐3	Glyma.11G107000	Amino acid transporters	1.6	48
	11	ss715612432	12	34480040	2.8 × 10^−5^	G/A	4.2		Glyma.12G183400	Acyl‐CoA reductase	1.6	12

a Position in base pairs for the peak SNP according to soya bean reference sequence (a2. v1) of Williams 82.

b The position of significant SNP is located in one of the QTL intervals (defined as physical position of associated markers) as reported previously (http://www.soybase.org/search/index.php?qtl=white mould).

c Candidate genes selected by RNA‐seq analysis as having the significant changes (FDR<0.05) in abundance between partially resistant and susceptible genotypes by comparisons of Log_2_ (fold change) of reads per kilobase per million (FPKM) around peak SNP.

d TP stands for time point in hours (hours postinoculation).

We compared the positions of the significant SNPs identified in this study with the positions of the QTL reported from previous bi‐parental and association mapping studies. Of the 11 loci we detected in the greenhouse trials, three overlapped with QTL previously identified from bi‐parental mapping studies (Table 2). Of the 16 loci we detected in the field trials, four reside within large intervals of QTL reported from previous bi‐parental mapping studies, and one (Chr. 15 at 12.3 Mb) locates within a small interval (from 12.2 to 13.2 Mb delimited by 2 SNPs) identified by a previous GWAS for white mould resistance in soya bean (Iquira *et al*., 2015).

As for the field trials data, 26 SNPs around 16 loci were significantly associated with DSI (Table 3 and Figure S4). These loci scattered across 12 chromosomes, and the peak SNPs at the identified loci explained approximately 45.6% and 51.7% of the total phenotypic variance in improved lines and PIs, respectively. In the panel of improved lines, the locus with the largest effect (*R* ^2^ = 8.2%) comprised of two SNPs (ss715605011 and ss715605026) covering 190 kb around 49.5 Mb on Chromosome 9. In the panel of PIs, the SNP showing the highest association (*P* value = 5.3 × 10^−6^) with DSI comprised of three SNPs (ss715624027, ss715624030 and ss715624031) covering 14 kb around 29 Mb on Chromosome 16. Only one locus (Locus #7) had a significant association with white mould resistance across both panels. One explanation for this unexpected result is that the two populations had a different genetic background and molecular mode of action underlying resistance. A NJ tree showed that the panels of PIs and improved lines formed highly differentiated populations (Wen *et al*., 2015).

**Table 3:** SNPs significantly associated with white mould resistance and a subset of candidate genes identified by RNA‐Seq from the field trials

Panel	Loci	SNP	Chr.	Position^a^	*P*	Allele	*R* ^2^(%)	QTL^b^	Subset of candidate genes^c^ based on RNA‐seq			
									Name	Annotation	Log2 (fold change)	TP d (hpi)
Improved lines	1	ss715583735	2	6447172	3.1 × 10^−5^	T/C	5.3		Glyma.02G073700	Aquaporin transporter	2.5	12
	2	ss715587841	4	3732457	3.4 × 10^−5^	C/T	5.3		Glyma.04G046600	Hypothetical protein	−2.1	12
		ss715587850	4	3752035	5.3 × 10^−5^	T/G	5.1					
		ss715587866	4	3797774	3.9 × 10^−5^	G/A	5.1					
	3	ss715587925	4	42372944	2.6 × 10^−5^	T/C	5.6		Glyma.04G184400	F‐BOX	1.5	12
		ss715588278	4	46104694	3.2 × 10^−5^	T/C	5.5					
	4	ss715590176	5	3924139	1.6 × 10^−6^	G/A	6.9		Glyma.05G044000*	Pectate lyase	1.6	12
	5	ss715601283	8	2789107	4.5 × 10^−5^	T/G	5.1		Glyma.08 g035900	Glycosyl hydrolase	1.6	12
	6	ss715605011	9	49559911	3.6 × 10^−5^	G/A	5.3	2‐18	Glyma.09G281900	O‐methyltransferase	2.2	48
		ss715605026	9	49749681	4.7 × 10^−7^	C/T	8.2					
	7	ss715624465	16	31915854	7.7 × 10^−6^	T/G	6.1		Glyma.16G158100	Glucuronosyltransferases	2.0	48
	8	ss715630705	18	43030373	1.2 × 10^−5^	T/C	6.0		Glyma.18G177400	Laccase	2.0	12
PIs	9	ss715590828	5	33208876	5.9 × 10^−5^	T/C	5.2	2‐1	Glyma.05G138800	Cytochrome b	1.8	12
	10	ss715596286	7	10514582	4.6 × 10^−5^	T/G	5.3	1‐2	–	–	–	–
	11	ss715607488	10	45331299	2.3 × 10^−5^	C/T	5.8		Glyma.10G221700	Solute carrier	1.5	12
	12	ss715618590	14	3852549	6.6 × 10^−5^	C/T	6.8		Glyma.14G049400	Protein binding	1.7	12
		ss715618599	14	3878273	3.7 × 10^−5^	A/G	5.9					
		ss715618604	14	3885274	1.5 × 10^−5^	T/C	6.0	8‐2				
	13	ss715620418	15	12264951	4.0 × 10^−5^	T/C	5.4	G.S	Glyma.15G147100	5′‐3′ exoribonuclease 3	4.2	12
		ss715620421	15	12278417	7.1 × 10^−5^	T/C	5.2					
	14	ss715624027	16	29081835	5.5 × 10^−5^	C/T	5.2		Glyma.16 g134000, Glyma.16G134400	SAM dependent carboxyl methyltransferase	1.8; 1.6	12
		ss715624030	16	29090022	2.3 × 10^−5^	T/G	6.0					
		ss715624031	16	29095909	5.3 × 10^−6^	G/A	7.7					
	7	ss715624900	16	31667215	6.1 × 10^−5^	C/T	5.2		Glyma.16G158100	Glucuronosyltransferases	2.0	12
	15	ss715636086	19	579512	6.1 × 10^−5^	G/A	5.2		Glyma.19G005800	Polyribonucleotide nucleotidyltransferase	1.6	12
	16	ss715634194	19	3498043	6.4 × 10^−5^	G/A	5.1		Glyma.19G026900	Plastocyanin‐like domain	1.9	12

a Position in base pairs for the peak SNP according to soya bean reference sequence (a2.v1) of Williams 82.

b the position of significant SNP is located in one of the QTL or GWAS (G.S) intervals (defined as physical position of associated markers) as reported previously (http://www.soybase.org/search/index.php?qtl=white mould).

c Candidate genes selected by RNA‐seq results as having the significant changes ((FDR<0.05) in abundance between partially resistant and susceptible genotypes by comparisons of fold change (log2‐transformed) of reads per kilobase per million (FPKM) around peak SNP.

d TP stands for time point (hours postinoculation).

### Characteristics of GWAS‐identified genes

Given that GWAS‐identified loci often fall within gene deserts or in regions with many equally plausible causative genes, it can be challenging to interpret GWAS signals biologically (Nica *et al*., 2010). Analysis of differential gene expression has been proposed as a promising approach to aid the interpretation (Emilsson *et al*., 2008). A previous study showed that genes that were found to have different expression patterns across varieties are most likely to be directly or indirectly related to specific susceptibility/resistance outcomes, while genes having differential expression across time points are most likely general responses of the plant to the infection, and may not lead to enhanced resistance (Calla *et al*., 2009). Therefore, we sequenced transcriptomes of four resistant and susceptible genotypes, and the following analyses were based on different expression patterns between the two genotypes.

Within GWAS‐identified loci based on greenhouse trials, a set of 58 genes were detected as having significant differential expression (FDR <0.05) between resistant and susceptible genotypes (Table S3). As for GWAS‐identified loci based on field trials, 49 genes were detected as having significant differential expression (Table S3). Of those genes, about half had more abundance in the resistant genotypes and half had more abundance in the susceptible genotypes. Although it is hard to arrive at reasonable conclusions about the exact mechanisms underlying white mould resistance based on these small sets of genes, both groups should be considered of great importance and be most likely candidates for improving resistance level in the partially resistant genotype. After assigning these genes to functional categories defined by Calla *et al*. (2009), the sum of genes in the categories ‘Defense’, ‘Signaling’ and ‘Unknown’ accounted for more than half the genes. Genes related to DNA/RNA processing, secondary metabolism protein synthesis and processing and membrane had lower percentages accounting for about 6% to 8%. Genes related to oxidative processes, cytoskeleton and cell wall accounted for only about 2% (Figure S5). Overall, the gene expression profiles were similar to some extent to those of PI 194639 (partially resistant soya bean genotype) seedlings in response to *S. sclerotiorum* infection (Calla *et al*., 2009). A comparison between the RNA sequences of those candidates from resistant and susceptible lines’ transcriptomes identified 32 nucleotide differences (24 single nucleotide polymorphisms (SNPs) and eight indels). Nine of the nucleotide differences from seven genes found result in an amino acid change in the predicted protein sequences (Table S4). Eight indels from eight genes create frameshift mutation.

As mentioned above, about half (58) of the differentially expressed genes were more abundant in the resistant line's transcriptome compared to the susceptible line's transcriptome. Among these up‐regulated genes, those encoding defence‐associated proteins, such as pectate lyase (*Glyma.05G044000*), phosphatase (*Glyma.09 g281900* and *Glyma.14G049600*) and methyltransferase (Glyma.16 g134700), were prominent (Table 2 and Table 3). Four NB‐ARC domains (*Glyma.09G062100*,* Glyma.16G135200*,* Glyma.16G135500* and *Glyma.16G159200*) were also significantly higher in abundance in the resistant lines at both time points studied. The NB‐ARC domain is believed to be a functional ATPase domain, and its nucleotide‐binding state is proposed to regulate activity of the resistance protein (van Ooijen *et al*., 2008). Moreover, there were two transferase‐related genes were induced within 12 h postinoculation (hpi), which encode acyltransferase (*Glyma.04G198000*) and a UDP‐glucosyltransferase (*Glyma.16G158100*) involved in secondary metabolism biosynthesis. The expression of this gene implies that a detoxification battle is being waged between host and pathogen (Zhao *et al*., 2009). Previous studies found that the secretion of oxalic acid of *S. sclerotiorum* can produce an unspecific toxin (Godoy *et al*., 1990; Zhao *et al*., 2015) in host plants. The oxalate exchanger‐related gene may play a role in the detoxification of oxalic acid. In our study, an oxalate exchanger‐related gene (*Glyma.06G106100*) was found located at a GWAS‐identified locus, and it was up‐regulated with log2 (fold change) = 2.8 (FDR <0.05) in the resistant line's transcriptome but had no significant change in susceptible line's transcriptome across time points. Furthermore, three additional oxalate exchanger‐related genes (*Glyma.07G218800*,* Glyma.13G087200* and *Glyma.19G159000*) exhibited elevated levels of transcripts in resistant line's transcriptome after inoculation with *S. sclerotiorum,* but did not overlap with GWAS‐identified loci. Future studies will focus on functionally validating effects of these genes, uncovering the molecular mechanisms of complex white mould resistance in soya bean.

### Marker‐assisted selection (MAS) and genomic selection for white mould resistance

**Figure 4:**
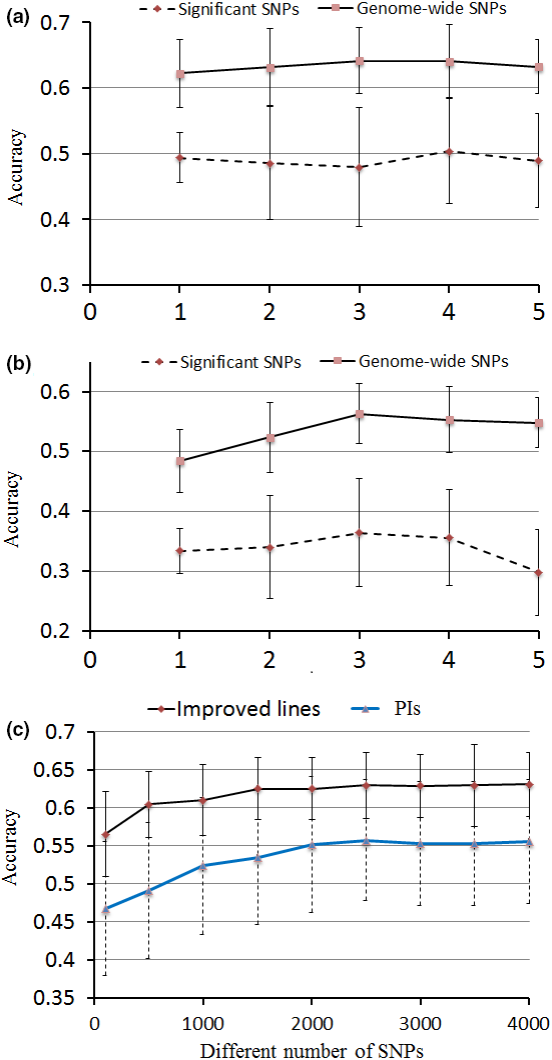
Mean accuracies of cross‐validation for prediction of DSI in two panels of soya bean germplasm. (a) Comparison of prediction accuracy of different fold between GS and MAS in improved lines (b) Comparison of prediction accuracy of different fold between GS and MAS in PIs. (c) Prediction accuracy with different number of SNP markers in GS for DSI. The prediction accuracy was the mean of fivefold estimated from fivefold cross‐validation with 100 replications within each fold.

Prediction accuracies of MAS using the loci identified via GWAS for DSI were investigated. For MAS by multiple linear regression (MLR) method, 12 and 14 SNPs identified from improved lines and PIs were investigated, respectively. At the same time, the prediction accuracies estimated from an equal number of randomly selected SNPs were used as a control. The prediction accuracies of MAS in the improved lines ranged from 0.47 to 0.51 (average of 0.50) for the 12 SNPs, which was 26% higher than that of the random SNPs (average of 0.37) (Figure 4a). Prediction accuracies for MAS in the PIs ranged from 0.29 to 0.36 (average of 0.34; Figure 4a, b), which was 24% higher than those for random SNPs (average of 0.26; Figure S6).

Based on the above analysis, it is clear that white mould resistance in soya bean is a complex trait and controlled by multiple genes with small effects. Our MAS model showed relatively low prediction accuracy for DSI. Moreover, it was recently suggested that MAS had failed to significantly improve complex traits (Heffner *et al*., 2009). Therefore, it was necessary to develop a genomic selection (GS) model for improving white mould resistance in soya bean. The same sets of phenotypic (DSI) and genotypic data used in the GWAS were used to assess the genomic prediction accuracy for white mould resistance through a fivefold cross‐validation. The prediction accuracies ranged from 0.62 to 0.64 for GS in the improved lines, whereas prediction accuracies ranged from 0.48 to 0.56 for GS in PIs (Figure 4a, b).

Although there are slight variations in prediction accuracies among the different folds (Figure 4a, b), the GS model overall outperforms the MAS model by ~20% in both populations. As it is important to determine the minimum number of markers for conducting GS in soya bean, differently sized SNP subsets were selected and the corresponding prediction accuracies were estimated. For both populations, there was no significant difference in prediction accuracies for DSI when 1500 SNPs (approximately 1 SNP for every 670 kb) were used versus when the full set of SNPs were used (Figure 4c). Note that the prediction accuracy in the improved lines remained >0.60 till the number of SNPs used for prediction dropped below 500.

With using a minimum of 1500 SNP markers, soya bean breeders are likely able to improve average prediction accuracy to 0.64, which is significantly greater than that of the conventional MAS approach (~0.41). The Illumina SoySNP6K iSelect BeadChip (Illumina, San Diego, CA), which consists of 5361 SNPs, has recently been developed for use specifically within soya bean breeding/research programmes (Ping *et al*., 2016; Wen *et al*., 2014). This BeadChip has established advantages in soya bean, including less bioinformatics analyses, robust and repeatable allele calling. As gBLUP was used in the present study, the higher prediction accuracy of GS in improved lines can be partially due to relative closer kinship among the sampled accessions (Figure 2c). Compared with previous GS studies in soya bean, the prediction accuracy of GS in this study was relatively lower than that of grain yield (0.64), seed weight (0.87) and soya bean cyst nematode (SCN) resistance (0.67) (Bao *et al*., 2014; Jarquín *et al*., 2014; Zhang *et al*., 2016). The higher prediction accuracy of GS in these previous studies could be due to higher heritabilities of the traits they investigated.

Collectively, GWAS has been proven very successful in discovering SNPs associated with complex traits, and now, it is imperative to explore their potential functional relevance. In this study, we successfully combined GWAS with RNA‐seq approaches to localize candidate genes underlying white mould resistance in soya bean. The present study can serve as a good reference for future studies on disease resistance in other plant species. Furthermore, we demonstrated that GS can be an effective tool to increase the efficiency of breeding for disease resistance in soya bean.

## Experimental procedures

### Sampling and genotyping

Two association panels were used in the present study. The first panel consisted of 405 accessions of soya bean PIs obtained from the USDA Germplasm Collection (hereafter named as PIs‐soybean‐405, ‘PIs’ for shortened form). These accessions were collected from multiple geographic origins including the United States, China, Japan, Korea, Kyrgyzstan and Russia. All of those accessions were selected to represent the variation and maintain the diversity of the collection, based on SNPs detected by the SoySNP50K BeadChip (Song *et al*., 2013) for material in maturity groups (MG) I, II and III. The second panel consisted of 962 improved lines released from 2007 to 2012 (hereafter named as Improved‐lines‐962‐MSU, ‘Improved lines’ for shortened form), which were chosen to represent a range of materials developed for North Central production area of the United States. Further information for each accession (selection criteria, commercial name and origin) is given in Table S5.

DNA samples from each accession were genotyped with SoySNP50 iSelect BeadChip (Illumina, San Diego, CA), which consists of 52 401 SNPs. The quality of each SNP was checked manually as previously reported by Yan *et al*. (2010). The SNPs with minor allele frequency (MAF) >5% and a missing data rate <20% were retained.

### White mould resistance evaluation in greenhouse and field trials

All soya bean accessions were grown in a greenhouse on the campus of Michigan State University, East Lansing. The experimental design was a randomized complete block design with two replicates. For each accession, six plants per replicate were evaluated at the V_3_ growth stage (Fehr *et al*., 1971) in pots. The *S. sclerotiorum* isolate 105HT provided by Dr. Glen Hartman (soya bean Pathogen Collection Center at the United States Department of Agriculture, Agricultural Research Service at the University of Illinois) was used for inoculations. The experiments were conducted in the winter of 2012 and 2013. The drop‐mycelium method developed by Chen and Wang (2005) was adopted to evaluate white mould resistance. Greenhouse day/night temperature was set at 24°C. Humidity was controlled by Trion Herrmidifier (model 707, Sanford, NC). Plants were individually rated with a scale of 0 to 4 (Figure S7) based on living node number 10 days after the inoculation.

Two subsets of soya bean lines were selected from two association panels, 278 PIs and 421 improved lines. To reduce the influence of lodging to white mould in field trials, all selected lines had lodged plants fewer than 25%. The two panels were evaluated for white mould resistance in a naturally infested white mould disease nursery at Montcalm, Michigan, during the growing seasons (May–October) of 2014 and 2015. Consistent heavy white mould disease symptoms had been observed historically in the disease nursery. Ninety seeds were planted in single‐row plots, 6 m long with 0.58 m row spacing, at a depth of 3.8 cm with three replications. Plots were rated for disease severity based on the rating system developed by Kim *et al*. (1999) at approximately the beginning of physiological maturity (R7; Fehr *et al*., 1971). All plants in the plots were individually rated with a scale of 0 to 3, where 0 = no symptoms, 1 = lesions on lateral branches only, 2 = lesions on the main stem but no effect on pod fill and 3 = lesions on main stem resulting in plant death and poor pod fill. A disease severity index (DSI) was calculated for each plot using the following formula:

$$\text{DSI}=\left(\sum (\text{rating of each plant})/3\times \text{total number of plants rated}\right)\times 100$$

Therefore, DSI ranges from 0 to 100 standing for no disease symptom to plant death. As the DSI data were collected from multiple years; best linear unbiased predictors (BLUPs) were used for the overall association analysis. The linear model for BLUP was *Y*_ijk_ **= *L*_*k*_ + *E*_*i*_ + *R* (*E*)_*ij*_ + (*L × E)*_*ik*_ + ε_*ijk*_, where *Y*_*ijk*_ is the observed phenotype for the *k* ^th^ line in the *j* ^th^ replicate of the *i* ^th^ environment; *L*_*k*_ is the random effect of the *k* ^th^ line; *E*_*i*_ is the random effect of the *i* ^th^ year; *R* (*E*)_*ij*_ is the random effect of the *j* ^th^ replicate in the *i* ^th^ year; (*E *× *L*)_*ik*_ is the random interaction effect of the *i* ^th^ year and the *k* ^th^ line, and ε_*ijk*_ is the error. The heritability estimates were calculated using variance components obtained by the BLUP linear model (Nyquist, 1991).

### Population structure and kinship analyses

Principal component and neighbour‐joining tree analysis were applied to infer population stratification. A pairwise distance matrix derived from a modified Euclidean distance for all polymorphic SNPs was calculated to construct neighbour‐joining trees using TASSEL 5.0 software (Bradbury *et al*., 2007). Principal component analysis was performed using TASSEL 5.0 based on 4549 SNPs with minor allele frequency (MAF) >20% and physical distance >60 kb. Kinship matrixes were calculated using centred IBS method (Endelman and Jannink, 2012) implemented in TASSEL 5.0 to determine relatedness among individuals based on the same sets of SNPs. TASSEL 5.0 was used to make all pairwise comparisons of alleles to calculate squared correlation coefficient (*r* ^*2*^) of alleles between markers. The extent of LD decay was measured as the chromosomal distance at which the average pairwise correlation coefficient (*r* ^*2*^) dropped to half its maximum value.

### Genome‐wide association analysis

A unified mixed model was used to perform GWAS with the control of both population structure and relative kinship. The MLM can be expressed as *y* + *X*α + *P*β + *K*μ + *e*, respectively, where *y* is the phenotypic value; α *is* the vector of SNP effects; β is the vector of population structure effects; μ is the vector of kinship background effects; *e* is the vector of residual effects; *P* is the PCA matrix relating *y* to β; *X* and *K* are incidence matrices of 1s and 0s relating *y* to α and μ, respectively (Zhang *et al*., 2010). The top five principal components were used to build the *P* matrix for population structure correction. Analyses were performed with the software TASSEL 5.0. False discovery rate (FDR) ≤0.05 was used to identify significant associations.

### Characterization of candidate genes based on RNA‐seq

To identify causative candidate gene around GWAS‐identified loci, the most resistant line (AG1703), the most susceptible line (V28N8RR) and resistance (R, AxN‐1‐55) and susceptible (S, Olympus) check were grown and inoculated with *Sclerotinia sclerotiorum* in greenhouse with the drop‐mycelium method (Chen and Wang, 2005). For each accession, the main stem tips (top 3 cm) were collected from two replicates at 12 and 48 h postinoculation hpi, respectively. Control samples (noninoculated, freshly cut stems from seedlings at 12 and 48 h hpi) were also collected. Samples were quickly packed into foil and frozen in liquid nitrogen within 10 s of collection.

Total RNA was isolated using the RNeasy Plant Mini Kit (Qiagen Inc., Valencia, CA) according to the manufacturer's instructions in conjunction with DNase treatment. The quality of total RNA was determined using RiboGreen^®^ RNA Assay Kit. Libraries were constructed and sequenced by MOGENE (Saint Louis, MO), and their sequencing reads were analysed as described by (Goettel *et al*., 2014). Tophat v2.0.1 (Trapnell *et al*., 2009) was run on each of the samples using the Williams 82 a2 v1 reference genome and transcriptome annotation from Phytozome v10 to guide the alignments. Cufflinks v2.2.1 (Roberts *et al*., 2011) was run on each sample bam to quantitate against reference transcript annotations only. Cuffmerge v1.0.0 (https://manned.org/cuffmerge/f06f1a10) was then used to produce the merged transcriptome file. Cuffdiff (v2.2.1; Trapnell *et al*., 2010) was used to produce normalized gene expression values in FPKMs (fragments per kilobase of exon per million fragments mapped), as well as an all by all differential expression analysis by combining replications. Differentially expressed genes in the specific paired sample comparisons were identified with the log_2_ fold change between the two samples and the *P*‐values given to the comparison along with the FPKMs for each of the two samples in the comparison.

### Genomic prediction and marker‐assisted selection model

A genomic best linear unbiased prediction (gBLUP) model was used to predict genomic estimated breeding values (GEBVs) of white mould resistance. The model for gBLUP is given by *y *=* *1_n_μ + Z*g* + *e,* where *y* is a vector of phenotypes, 1_n_ is a vector of ones, μ is the mean, Z is a design matrix allocating records to genetic values, *g* is a vector of additive genetic effects for an individual, and *e* is a vector of random normal deviates σ^2^. Analyses were performed with the software TASSEL 5.0.

As for the MAS model, MLR was employed to predict DSI (Zhang *et al*., 2016). The Pearson correlation coefficient between the observations and the cross‐validated GEBVs was used to determine the accuracy. To compute the accuracy, we used a fivefold cross‐validation. Each phenotypic data set was randomly divided into five equal parts. The GEBVs for each fold were later predicted by training the model on the four remaining folds.

To investigate the prediction accuracies with different number of markers, nine subsets of SNPs that were evenly distributed across the genome were selected. The subsets sizes were 100, 500, 1000, 1500, 2000, 2500, 3000, 3500 and 4000 corresponding to interval distance of 10.0 Mb, 2.0 Mb, 1.0 Mb, 0.67 Mb, 0.5 Mb, 0.4 Mb, 0.3 Mb, 0.28 Mb and 0.25 Mb, respectively. Each subset was then used as the genotype matrix to perform fivefold cross‐validation across both two panels.

## Supporting information


**Figure S1** Histograms showing the distributions of phenotypic data observed in greenhouse trials.
**Figure S2** Histograms and box‐plots showing the distributions of phenotypic data observed in field trials.
**Figure S3** Quantile‐quantile (QQ) plot of MLM for living node and DSI in two panels.
**Figure S4** Manhattan plots of MLM for DSI in improved lines (a) and PIs (b).
**Figure S5** Functional category annotations for candidate genes and their respective percentages identified via GWAS as significantly associated with white mould resistance.
**Figure S6** Comparison of predication accuracy between significant SNP and randomly selected SNP.
**Figure S7** Scale used for phenotyping white mould disease severity (DS).
**Table S1** Correlation analysis of DSI and agronomic traits in improved lines and PIs.
**Table S2** Distribution of accessions in each subgroup based on genetic distance in improved lines and PIs.

**Table S3** Candidate genes showing statistically significant induction in response to *Sclerotinia sclerotiorum* inoculation among genotypes tested.

**Table S4** Nucleotide differences found between resistant and susceptible genotypes result in an amino acid change at GWAS‐hit loci.
**Table S5** Soya bean germplasm accessions analyzed in this study.
