# Supplementary material for: Integrating GWAS and gene expression data for functional characterization of resistance to white mould in soya bean
Source: Plant Biotechnol J. 2018 May 7;16(11):1825–35. doi: 10.1111/pbi.12918 (PMC6181214; doi:10.1111/pbi.12918)
Supplement: Supplementary file 1 — Figure S1 Histograms showing the distributions of phenotypic data observed in greenhouse trials. Figure S2 Histograms and box‐plots showing the distributions of phenotypic data observed in field trials. Figure S3 Quantile‐quantile (QQ) plot of MLM for living node and DSI in two panels. Figure S4 Manhattan plots of MLM for DSI in improved lines (a) and PIs (b). Figure S5 Functional category annotations for candidate genes and their respective percentages identified via GWAS as significantly associated with white mould resistance. Figure S6 Comparison of predication accuracy between significant SNP and randomly selected SNP. Figure S7 Scale used for phenotyping white mould disease severity (DS). Table S1 Correlation analysis of DSI and agronomic traits in improved lines and PIs. Table S2 Distribution of accessions in each subgroup based on genetic distance in improved lines and PIs. [file PBI-16-1825-s001.docx]

**
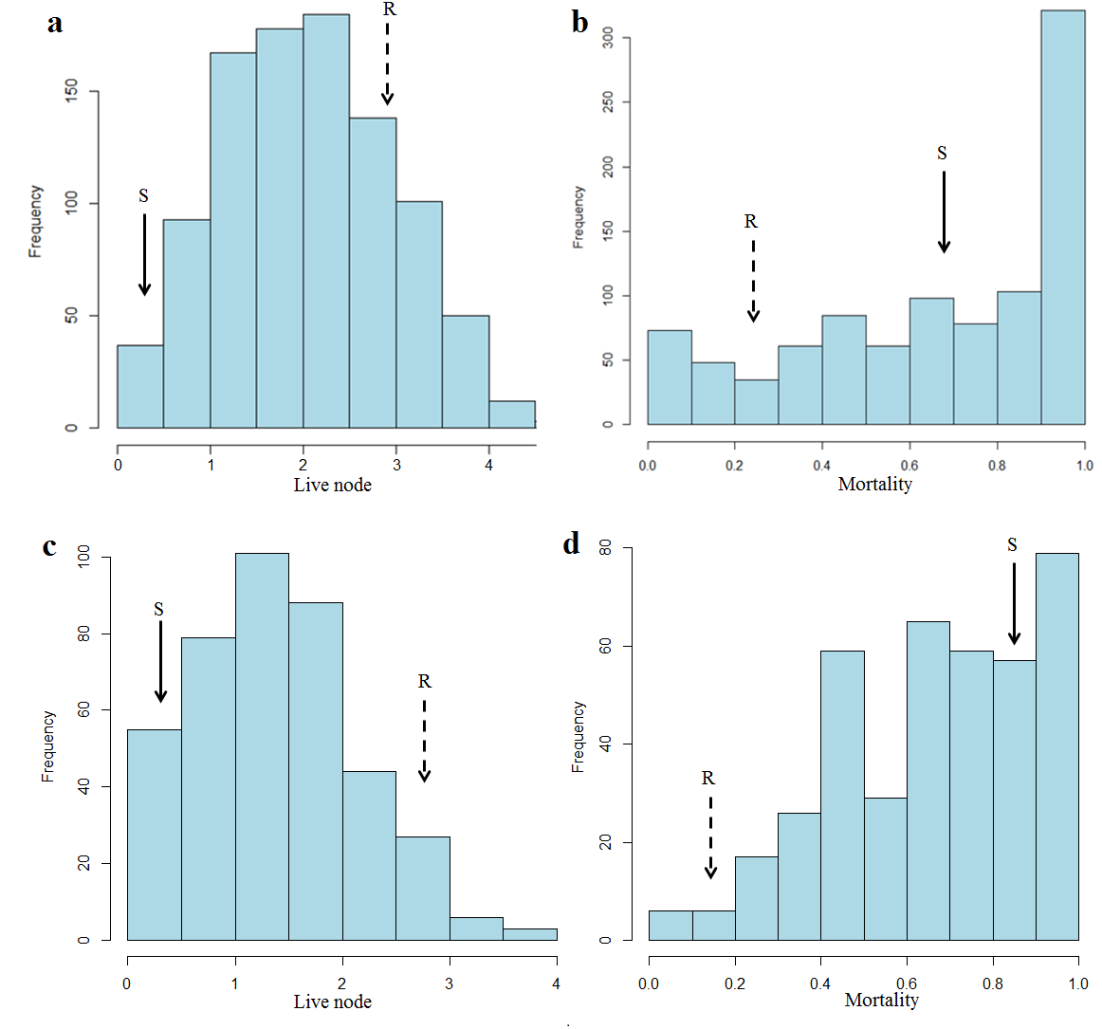
**

**Supporting Figure S1.** Histograms showing the distributions of phenotypic data observed in greenhouse trials. Distribution of living node number in improved lines (a) and PIs (c). Distribution of mortality in improved lines (b) and PIs (d).; R, resistant check (AxN-1-55); S., susceptible check (Olympus)

**
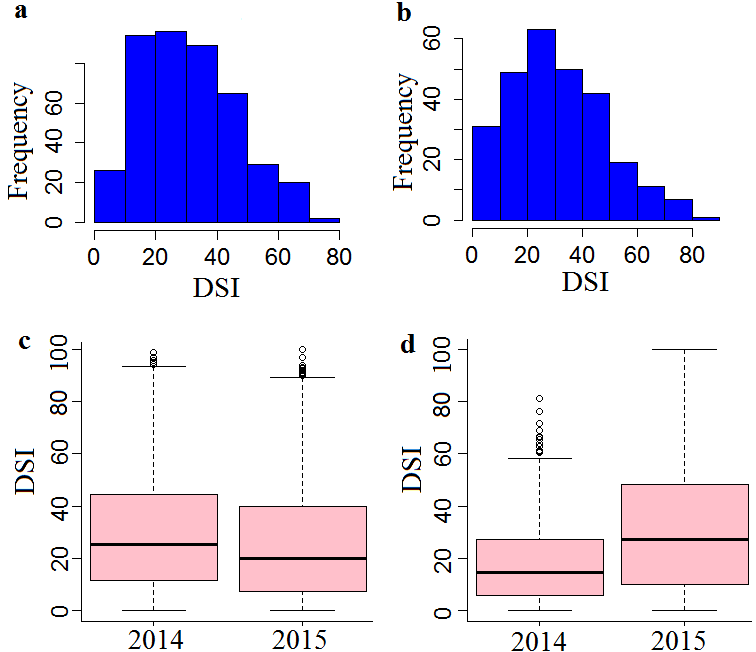
**

**Supporting Figure S2: Histograms and box-plots showing the distributions of phenotypic data observed in field trials . Distribution of DSI in improved lines (a) and PIs (b). Box-plot of DSI in improved lines (c) and PIs (d).**

**
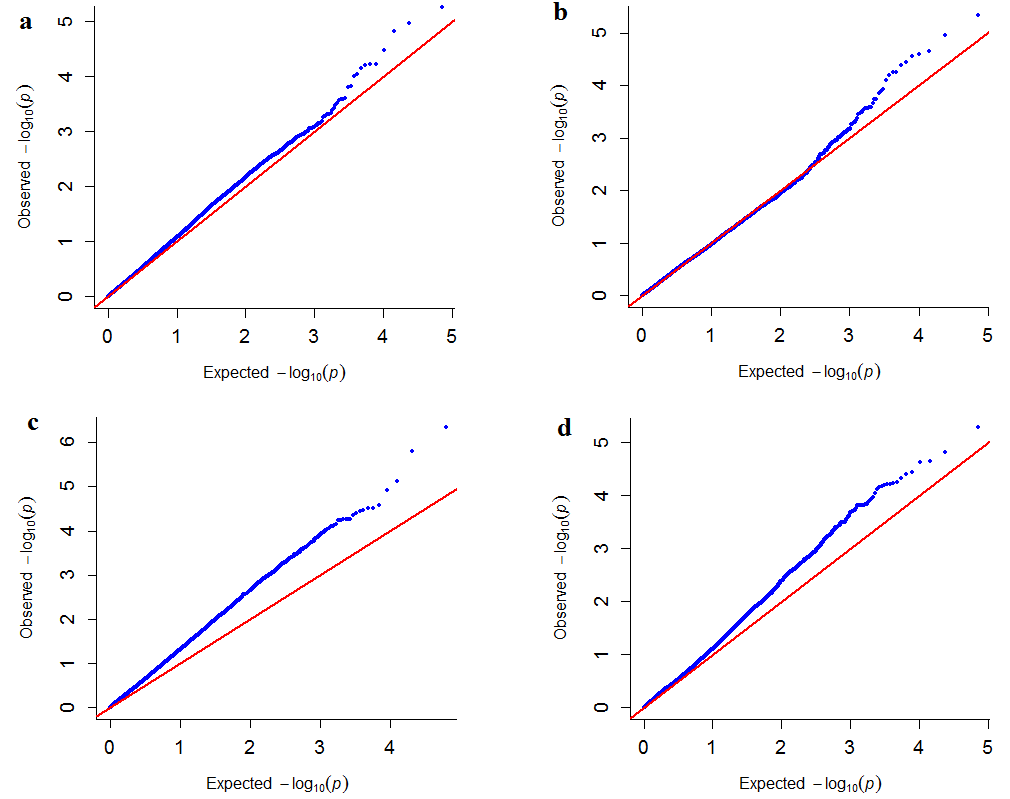
**

**Supporting Figure S3: Quantile-quantile (QQ) plot of MLM for living node and DSI in two panels. (a)** Quantile-quantile (QQ) plot of MLM for liveing node in improved lines. (b) Quantile-quantile (QQ) plot of MLM for liveing node in PIs. (c) Quantile-quantile (QQ) plot of MLM for DSI in improved lines. (d) Quantile-quantile (QQ) plot of MLM for liveing node in PIs.

**
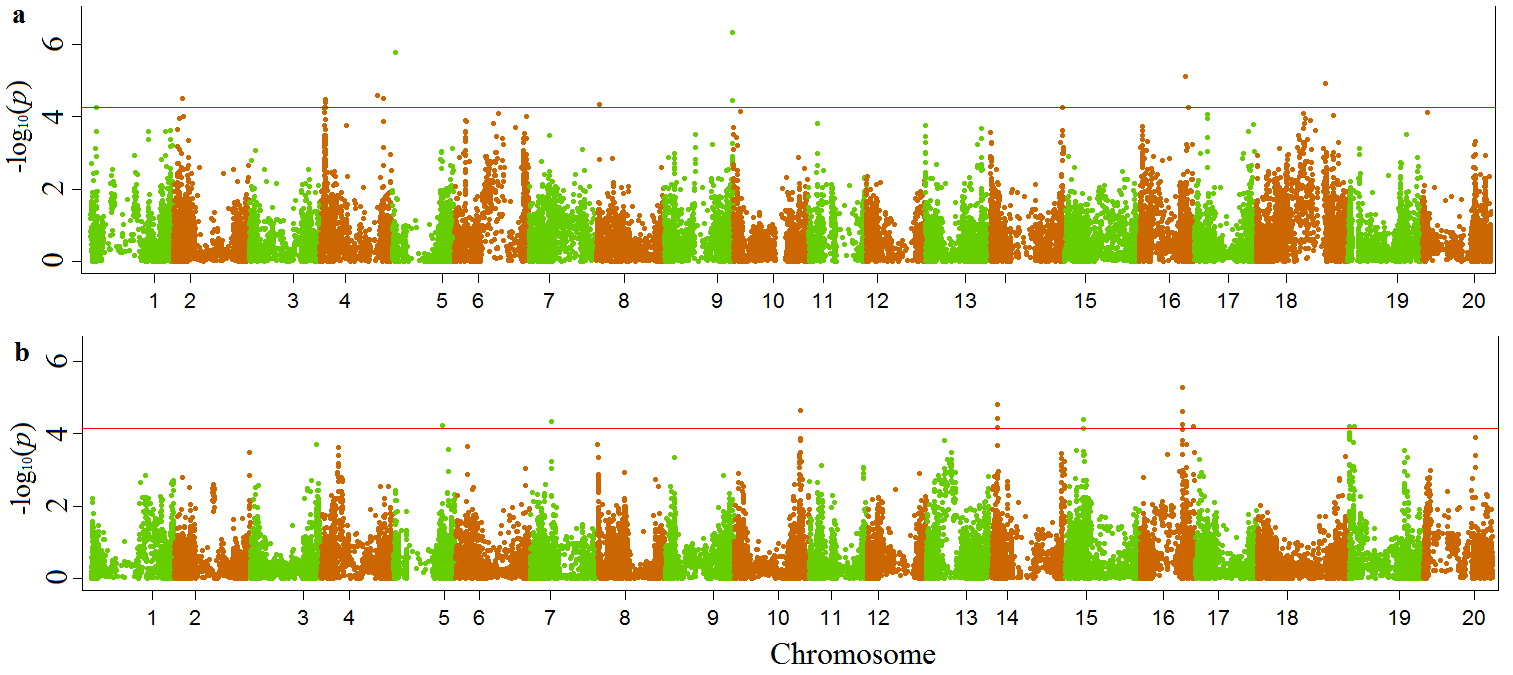
**

**Supporting Figure S4: Manhattan plots of MLM for DSI in improved lines (a) and PIs (b)**. The − log10 P-values from a genome-wide scan are plotted against the position on each of the 20 chromosomes. The horizontal red line indicates the genome-wide significance threshold (FDR < 0.05)

**
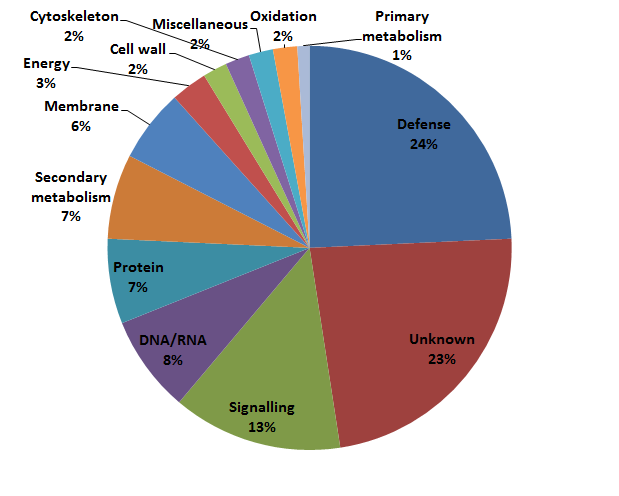
**

**Supporting Figure S5. Functional category annotations for candidate genes and their respective percentages identified via GWAS as significantly associated with white mold resistance**

**
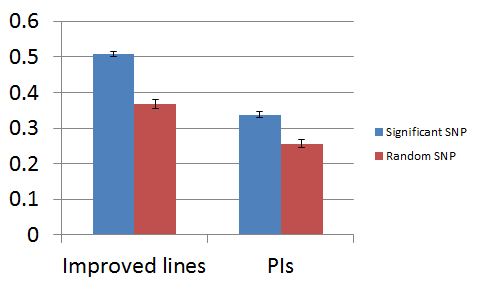
**

**Supporting Figure S6: Comparison of predication accuracy between significant SNP and randomly selected SNP**

**
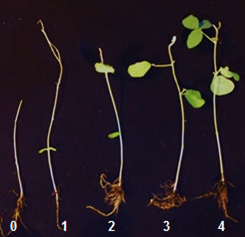
**

**Supporting Figure S7: Scale used for phenotyping white mold disease severity (DS).**

**Supporting Table S1 Correlation analysis of DSI and agronomic traits in improved lines and PIs.**

| Panel | | Trait | DSI_2015 | DSI_2014 | DSI_2013 | MT | Lodging |
| --- | --- | --- | --- | --- | --- | --- | --- |
| Improved lines | | DSI_2015 | - |  |  |  |  |
|  | | DSI_2014 | 0.61** | - |  |  |  |
|  | | DSI_2013 | 0.41** | 0.52** | - |  |  |
|  | | Maturity | -0.04 | -0.01 | -0.09* | - |  |
|  | | Lodging | 0.10* | 0.11* | 0.04 | 0.24** | - |
|  | | Living node | -0.12* | -0.22** | -0.15* | 0.03 | -0.1* |
| PIs | |  |  |  |  |  |  |
|  |  | | Lodging | Flowering time | Maturity | DSI_2014 | DSI_2015 |
|  | Lodging | | - |  |  |  |  |
|  | Flowering Time | | 0.22** | - |  |  |  |
|  | Maturity | | 0.16** | 0.64** | - |  |  |
|  | DSI_2014 | | 0.17* | -0.18** | -0.01 | - |  |
|  | DSI_2015 | | 0.22** | 0.10 | 0.07 | 0.39** | - |
|  | Living node | | -0.07 | -0.07 | -0.01 | -0.03 | -0.06 |

***Significant at P < 0.05, **Significant at P < 0.01**

**Supporting Table S2 Distribution of accessions in each subgroup based on genetic distance in improved lines and PIs**

| Population |  | | | Clusters of NJ tree | | | | | | | |  |  |
| --- | --- | --- | --- | --- | --- | --- | --- | --- | --- | --- | --- | --- | --- |
| PIs |  | 1(black) | | 2(green) | | 3(dark green) | | 4(purple) |  |  |  |  |  |
|  | NC | 19 | | 34 | | 14 | | 22 |  |  |  |  |  |
|  | HHC | 0 | | 3 | | 2 | | 18 |  |  |  |  |  |
|  | SC | 0 | | 0 | | 0 | | 1 |  |  |  |  |  |
|  | Korea | 1 | | 3 | | 5 | | 12 |  |  |  |  |  |
|  | Japan | 2 | | 2 | | 4 | | 14 |  |  |  |  |  |
|  | Far East | 2 | | 19 | | 5 | | 2 |  |  |  |  |  |
|  | NA | 0 | | 3 | | 45 | | 5 |  |  |  |  |  |
|  | other | 7 | | 16 | | 5 | | 8 |  |  |  |  |  |
| Improved lines | *χ*^2^ test | *χ*^2^= 173.8 >*χ*^2^_0.01,21_ =38.9 , *P*<0.001 | | | | | | | |  |  |  |  |
|  |  | Clusters of NJ tree | | | | | | | | | |  |  |
|  |  | 1(red) | 2(purple) | | 3(brown) | 4(blue) | 5(green) |  | | | |  |  |
|  | MG I  MG II  MG III | 4 | 1 | | 0 | 93 | 5 |  | | | |  |  |
|  |  | 30 | 14 | | 0 | 147 | 27 |  | | | |  |  |
|  |  | 27 | 13 | | 8 | 42 | 10 |  | | | |  |  |
|  | *χ*^2^ test | *χ*^2^= 77.3> *χ*^2^_0.01,8_ = 20.9, *P*<0.001 | | | | | | | |  | |  |  |

NC: North China, HHC: Huanghuai China, SC:South China, NA: North of America, MG, maturity group

**Supporting Table S4** Nucleotide differences found between resistant and susceptible genotypes result in an amino acid change at GWAS-hit loci

| Gene | Position | Resistant genotypes | |  | Susceptible genotypes | |  | Amino acid change | |
| --- | --- | --- | --- | --- | --- | --- | --- | --- | --- |
|  |  | R._most | R._Check |  | S._most | S._check |  | R. | S. |
| *Glyma.04G180900* | 509 | G/G | G/G |  | A/G | A/G |  | His | Arg |
| *Glyma.06G107800* | 1490 | G/G | G/G |  | T/G | T/G |  | Leu | Arg |
| *Glyma.08G035900* | 377 | T/A | T/A |  | A/A | A/A |  | Phe | Tyr |
| *Glyma.09G062100* | 82 | A/G | A/G |  | G/G | G/G |  | Lys | Glu |
|  | 1094 | G/G | G/G |  | ./. | ./. |  | Gln | Arg |
|  | 1399 | C/C | C/C |  | ./. | ./. |  | Trp | Arg |
| *Glyma.10G214400* | 134 | C/C | C/C |  | A/C | A/C |  | Tyr | Cys |
| *Glyma.15G147100* | 167 | T/C | T/C |  | C/C | C/C |  | Leu | Pro |
| *Glyma.19G005900* | 112 | T/T | T/T |  | C/C | C/C |  | Ser | Pro |

R., resistant genotype; S., susceptible genotype; R_most, most resistant line (AG1703); R_check, resistant check (AxN-1-55); S_most, most susceptible line (V28N8RR); S_check, susceptible check (Olympus); ./., deletion mutation
